# Absolute Calibration for Cyclic Voltammetry from the Solution-Phase Ionisation of Ferrocene

**DOI:** 10.1021/acselectrochem.5c00382

**Published:** 2026-01-20

**Authors:** Tomi K. Baikie, Jonathon R. Harwell, Iain D. Baikie, Eli Zysman-Colman, Ifor D. W. Samuel, Graham A. Turnbull

**Affiliations:** † Organic Semiconductor Centre, SUPA, School of Physics and Astronomy, 7486 University of St Andrews, St Andrews KY16 9SS, U.K.; ‡ Cavendish Laboratory, 2152 University of Cambridge, Cambridge CB3 0EH, U.K.; § Research Laboratory of Electronics, Massachusetts Institute of Technology, Cambridge, Massachusetts 02139, United States; ∥ KP Technology Ltd, The Old Foundry, Burn Street, Wick KW1 5LE, U.K.; ⊥ Organic Semiconductor Centre, EaStCHEM School of Chemistry, University of St Andrews, St Andrews KY16 9ST, U.K.

**Keywords:** ferrocene cyclic, voltammetry photoemission, yield spectroscopy Fermi scale

## Abstract

Accurate determination of the energy levels of materials is crucial to many fields of science and technology, including electronics, catalysis, and energy generation and storage. The frontier molecular orbital levels of molecules are commonly inferred from their oxidation and reduction potentials measured in solution using voltametric techniques, which are reported versus a standard, typically an internal one such as a ferrocenium/ferrocene (Fc^+^/Fc) redox couple. At present, however, multiple reference electrode scales are used across the literature, leading to discrepancies of up to 0.3 eV. Here, we report an absolute energy level measurement for (Fc^+^/Fc) in acetonitrile solution. Specifically, we determined the adiabatic ionisation energy of ferrocene in acetonitrile solution to be 4.94 ± 0.05 eV using ambient pressure photoemission spectroscopy. By comparing the energy-dependence of photoemission from different solution concentrations with a model for photoemission from solution, we confirm that we measure the adiabatic ionisation energy and that liquid surface barrier effects are minimal. This value is consistent with one of several conflicting reference values used in the literature. The result therefore provides a benchmark value for the Fc^+^/Fc internal reference, widely used for the conversion of voltammetry data to the absolute energy scale.

## Graphical abstract

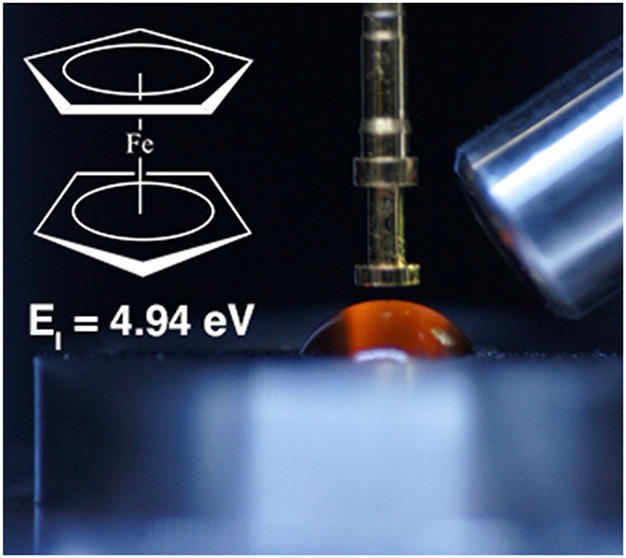

An accurate determination of the energy of the highest occupied molecular orbital (HOMO) of organic semiconductor materials is needed for the optimized design of optoelectronic and photonic devices such as organic light-emitting diodes (OLEDs), organic field effect transistors, organic photovoltaics (OPVs), and systems for catalysis, energy storage, and solar fuels.^1–12^ With the growing importance of organic electronics and energy materials, it is essential to have reliable experimental methods to determine the energies of their frontier molecular orbitals. Currently, these are routinely determined from solution-state measurements, such as cyclic voltammetry (CV) from which an estimate of the HOMO and lowest unoccupied molecular orbital (LUMO) energies are inferred from electrochemical redox potentials. Oxidation and reduction potentials can be related to HOMO/LUMO energies when the former are referenced to ferrocenium/ferrocene (Fc^+^/Fc). This conversion is predicated on an accurate measure of the ionization energy of this reference, which has historically been measured in the solid state. This presents problems as (1) the use of a measurement in the condensed phase compared to one in dilute solution may not be appropriate; and (2) redox potentials are sensitive to many circumstantial factors such as the choice of solvents, electrodes, or the electrolyte used in the experiment, which gives rise to uncertainties as high as 0.3 eV.^1,13–16^ In this letter, we attempt to resolve these ambiguities and directly relate the internal reference couple to the absolute energy scale using only solution-state measurements.

Photons carry discrete amounts of energy as an intrinsic property independent of external observers.^17^ This property allows photoemission spectroscopy (PES) to make an absolute measurement of energy levels. Techniques such as ultraviolet photoelectron spectroscopy (UPS), are routinely conducted under high vacuum and are therefore normally restricted to solids. While the ionisation energy (IE) of liquid water has been determined with UPS,^18^ the stipulation for high vacuum measurements makes such experiments for solutions challenging and typically involves measurements of a liquid jet at micron-scale dimensions.^19,20^ Consequently, there are few reported measurements of organic molecules dissolved in these aqueous liquid jets.^21,22^

A practical approach to measuring photoemission from liquids is to hold the sample at ambient pressure. Such measurements, known as ambient pressure photoemission spectroscopy or photoemission yield spectroscopy (PYS) in air, have previously been used to accurately measure the ionization energy (IE) of solid metals and semiconductors with good agreement to literature values determined by XPS and UPS.^23,24^ Photoemission measurements of liquids have long been shown to be possible under ambient conditions, initially demonstrated by adaptations of Millikan’s seminal oil drop experiment^25,26^ and later advanced by Delahay,^27,28^ and Brodskii,^29,30^ amongst others.^31^

In the present work, we adapt PYS instrumentation (a Kelvin Probe,^32,33^ see Methods section for details) to carry out photoemission spectroscopy at ambient pressure and temperature to determine the absolute HOMO level of molecules in solution. The Fc^+^/Fc redox couple is the IUPAC recommended internal reference since the cyclic voltammogram of Fc^+^/Fc is completely reversible regardless of scan rate.^1,34–37^ Acetonitrile is a common solvent in CV measurements due to its large electrochemical window and its ability to co-dissolve a wide range of analytes at mM concentrations with respect to the electrolyte salt, which is required to aid charge transport between the electrodes in the cell. Thus, the absolute measurement of Fc^+^/Fc in acetonitrile has broad implications by benchmarking cyclic voltammetry data to the absolute energy scale.

Figure 1 gives an overview of our study and shows the UV-Visible absorption spectrum of ferrocene in acetonitrile. Consistent with other reports, ferrocene exhibits three broad bands at <250 nm, ∼330 nm, and ∼450 nm. The high-energy bands correspond to π–π* transitions of the cyclopentadienyl rings. The origin of the electronic transitions at 332 nm is still not clear from the literature, although the molecular orbitals of the rings are involved in this electronic transition.^38^ The band situated in the visible range at 442 nm is assigned to a Laporte-forbidden *d*–*d* transition localized on the iron metal center. Importantly, acetonitrile shows no significant absorbance in the relevant spectral window for our PYS experiments (up to 5.5 eV). As acetonitrile is transparent to photons of energies below 6.5 eV, the absorbance of the solvent is not expected to significantly limit the depth of photoemission.

**Figure 1:**
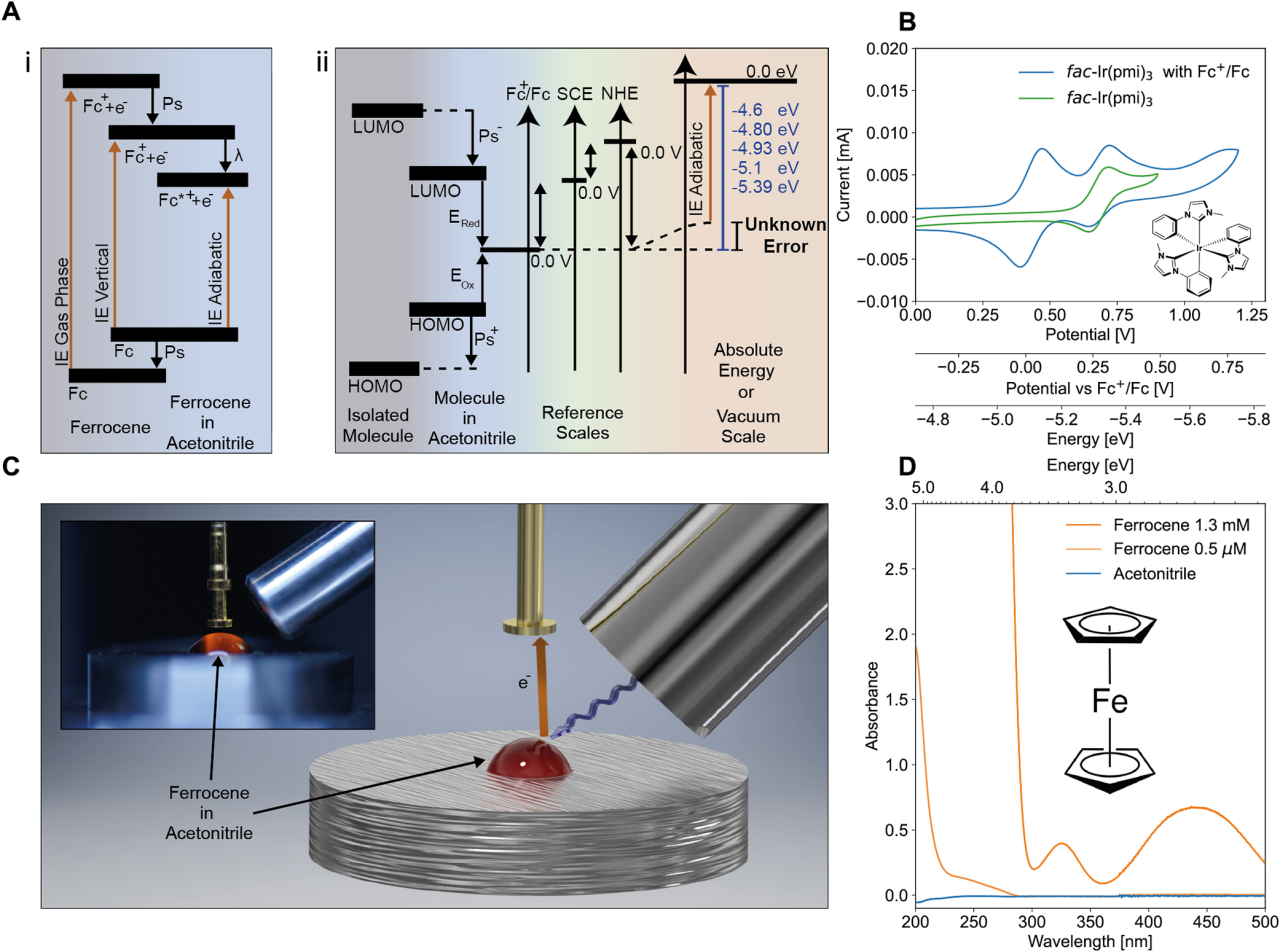
(A­(i)) Transition energies in a photoemission experiment of ferrocene in the gas phase,and in solution, where the vertical and adiabatic transitions are separated by the reorganisation energy (λ). Fc^+^ refers to the state after solvent reorganization. (A­(ii)) Energy levels of molecules and their measurement on the Fermi scale using cyclic voltammetry (CV). When dissolved in solution, solvent-dependent polarisation and solvation effects (Ps^–^, Ps^+^) shift the LUMO and HOMO closer together. CV measures the electrochemical potentials, (*E*_red_, *E*_ox_) and relates them to the ferrocene/ferrocenium (Fc/Fc^+^) redox couple (in green). Two of the typical scales are the normal hydrogen electron (NHE) or the saturated calomel electrode (SCE). These reference electrodes are themselves related to the Fermi scale where 0 eV is the vacuum level. (B) Example CV measurement for *fac*-Ir­(pmi)_3_:^57^ the voltammogram of *fac*-Ir­(pmi)_3_ is measured (green line) and then remeasured (blue line) after ferrocene is added to the solution to allow the voltage to be re-referenced with the *E*_1/2_^ox^ of Fc/Fc^+^ set to 0 V relative to a saturated calomel electrode (SCE). Using the translational values, the Fc^+^/Fc redox couple can be scaled to the Fermi scale, and the HOMO energy of *fac*-Ir­(pmi)_3_ recovered. (C) Schematic of liquid photoemission measurements, with photograph of apparatus shown inset. The sample is illuminated by a tunable light source. Upon photoemission, the negative charges drift towards the positively biased tip, as depicted by the orange arrow, where they are collected. (D) Absorption spectra of ferrocene in acetonitrile solution (orange), and acetonitrile solvent (blue).

The energy of the HOMO of a molecule is typically inferred from CV measurements in solution. *E*_HOMO_, is calculated by measuring the onset of the oxidation, *E*_onset_^*ox*^, relative to the *E*_1/2_ of Fc^+^/Fc, which is set to 0 V, (e.g., in Figure 1A) and applying the relation

1 $$E_{\mathrm{HOMO}}=-E_{\mathrm{onset}}^{\mathrm{ox}}-C\left[\mathrm{eV}\right]$$

where *C* is a constant used to map the Fc/Fc^+^ reference redox potential to the vacuum scale. Some authors use *E*_1/2_^ox^ rather than the onset for the frontier orbital energies, although this is discouraged,^1,40^ and becomes even more frustrated for irreversible systems where it is no longer possible to explicitly define *E*_1/2_^ox^. Further complications and errors arise since electrochemical data are generally referenced to an external electrochemical scale, such as the normal hydrogen electrode (NHE) or the saturated calomel electrode (SCE), commonly used in solar cell research and OLED research, respectively, although other scales exist.

The wide range of uncertainty in the absolute value relative to the vacuum scale of the Fc^+^/Fc redox couple internal reference is due to the various relative methods that have been previously used to attempt to determine its absolute value as depicted in Figure 1.^1–5,13,37,41–51^ Bard and Faulkner report −4.5 eV (or −4.4 eV) to be 0.0 V vs NHE[Fn fn1],^2^ whereas Hansen and Hansen^4^ report −4.456 eV, Trasatti repor −4.4 eV,^3^ and Kelly et al.^52^ report −4.52 eV, amongst others.^1^ Ferrocene’s redox couple has been reported to be 0.38 V vs SCE,^37^ 0.40 V vs SCE,^13^ and 0.41 V vs SCE^1^ in acetonitrile. Roberts and Bullock^53^ established the value for Fc^+^/Fc vs SHE in acetonitrile as 0.028 V. The standard potential of SCE has been determined to be 0.24 V vs NHE^2^ or 0.25 V vs NHE.^37^ Using the 0.40 V vs SCE value for the *E*_ox_ of Fc thus suggests the IE of Fc^+^/Fc in acetonitrile to be around −5.1 eV. Some report an absolute value of −5.39 eV for the Fc^+^/Fc couple.^1,41–43^ and others report −4.75 eV.^1^ Additionally, orbital energies can be calculated directly from electrochemical data, neglecting solvent and electrolyte effects, to determine a ferrocene redox couple of −4.8 eV.^5,15,1^ The −4.8 eV value is a widely used reference for scaling to the vacuum scale.^37,52–55^ Computational methods also guide understanding of the orbital levels for Fc but sometimes suffer from large errors in the estimation of these (particularly for the LUMO), and careful choices are required for the functional and basis set (see SI Section 1 for discussion).^1,15,44,56^ Of particular relevance to our approach is the computed redox potential of Fc/Fc^+^ of – 4.93 eV by Makos et al.^55^ Generally, either −5.1 eV or −4.8 eV is used throughout the literature^1^ to scale electrochemical results obtained by CV to the vacuum scale to infer HOMO (and LUMO) levels (we provide a more complete account of the above values in SI Section 1).

Such a ∼0.3 eV uncertainty in the reference energy has direct implications in the choice of materials used in devices such as OLEDs and OPVs. In such devices a 0.3 eV error could form a substantial barrier to charge injection or transport.^23^ Thus, comparing the HOMO level of organic semiconductor materials from CV measurements with the expectation of predicting possible performance may prove, and has proven to be, misleading.^1,16,35^ For example, for applications attempting to match two contacts or generate junctions intended for terrestrial energy capture, HOMO and LUMO levels mapped to the absolute energy scale would give a propagated uncertainty of ∼60% relative to the useful energy output.

In our experimental setup, see Figure 1C, a monochromated UV light source, tunable from 3.6 to 7.0 eV, illuminates the sample in air at ambient pressure. In this environment, the mean free path of the photoelectrons (∼1 μm) is too short to reach the detector, and momentum information on the photoelectron is lost.^23^ This contrasts with XPS and UPS, where the momentum of photoelectrons is recorded. In PYS, all photoelectrons are measured, hence the measurement operates as a sum over all electron momenta that may escape. At the onset, the exact band edge is obscured by thermal excitation of electrons and just beyond the onset the yield is related to the density of states, which is assumed to vary relatively slowly at the threshold. It is well established that a power law may be applied to PYS measurements with an extrapolation to the IE to account for these effects.

We approach the problem, broadly following the work of Brodskii,^29^ Delahay,^27,28,58^ and Gurevich^30,31^ and we acknowledge the criticism of their respective approaches amongst each other. Figure 2A outlines a summary schematic of the photoelectron steps we model, in detail in SI Section 2, but briefly here, we assume photoionization of dissolved ferrocene occurs within ∼100 fs and leads to a free electron (sometimes termed “dry”). If the electron becomes solvated (sometimes termed “bound”), we assume it does not contribute to the photocurrent. As solvation distance is much less than the penetration depth of the light, the solvation distance enforces surface sensitivity of the technique. We characterise non-elastic scattering, elastic scattering away from the surface, electron capture, stabilization, and other solvent effects as an exponential damping term on free electrons. Once the free electron reaches the interface, it may be transmitted or reflected. We reduce the problem to the one-dimensional Schrödinger equation utilising the orthogonality of spherical harmonics. Near the threshold onset energy, we only consider the harmonics with momentum largely perpendicular to the surface. Such an approach allows us to determine the form of the photocurrent as a function of input energy that accounts for the liquid/gas barrier and the image force experienced by an electron in the gas phase.

**Figure 2:**
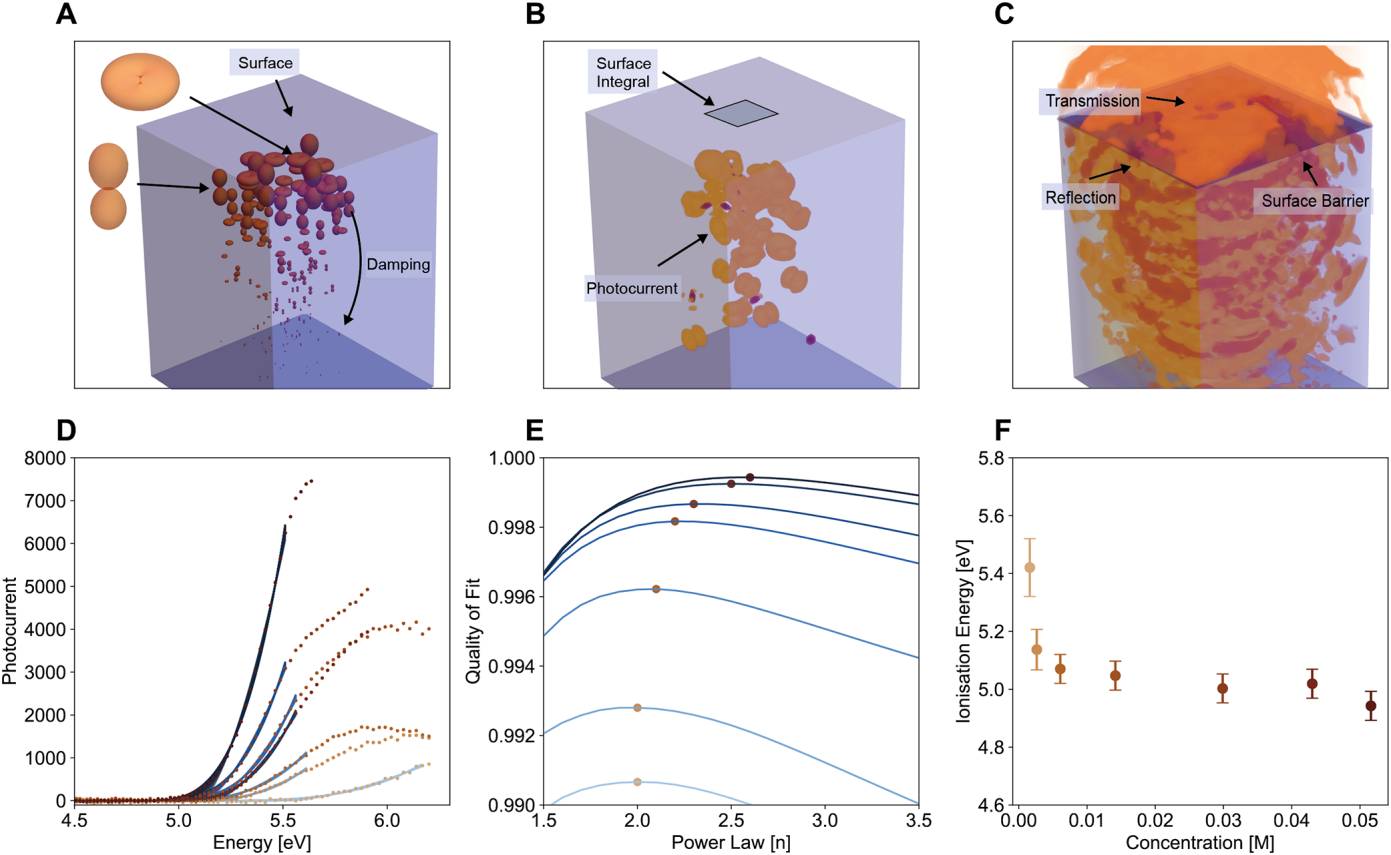
(A–C) Ferrocene photoelectron emission modelled with spherical harmonic emitters. Damping reduces the contribution of deeper molecules. An integrated transmitted wave normal to the solution gives the photocurrent. Some of the photocurrent is reflected back into the solution. (D) Photoemission yield spectra from ferrocene solutions in acetonitrile. Measured photoelectron currents (dots) as a function of photon energy, with power law fits for a subsection of values in the linear regime from 0.5 eV from the onset value. (E) Quality of fit as a function of power law. (F) Onset energies determined with a power law with best fit for photoemission as a function of concentration. The shade of datapoints in (D–F) directly corresponds to concentrations in (F).

The properties of the solution barrier are rather complex, as is the nature of the image force felt by the electron upon leaving the solution. Experimental surface potential measurements suggest that acetonitrile has a small surface potential at the solvent-gas interface barrier that gives rise to a barrier on the order of meV,^59^ see SI Section 3 for details. Here, we only assume some form of barrier where the electron may be either reflected back into the solution (and so does not contribute to the photocurrent) or transmitted into the gas phase. In the latter case, the electron will be subject to some form of an image force.

The photoelectron yield follows

2 $$Y\propto {\left(h\nu -\mathrm{IE}\right)}^{n}$$

where *Y* is the photoemission yield and *h*ν is the photon energy. A photoelectron experiences an image force when leaving a metal, which falls with distance from the emitting surface. When Coulomb-like image forces are included, *n* = 2 and the yield as in eq 2 follows a trend identical to that of well-established Fowler theory.^45,60,61^ The limit of a diminished image force, where *n* = 5/2, has been identified empirically as the best fit for photoelectron emission from solution.^27,28,58^ Here we make no determination on the physical origins of such electron screening in solutions (although we discuss plausible mechanisms in SI Section 2) and fit eq 2 for n∈R , which captures contributions from any change in the surface potential as a function of concentration and image force when *n* deviates from 5/2.

The PYS threshold energy can thus be established using eq 2, and we now address the question of which thermodynamic ionisation energy (vertical or adiabatic) is recovered. In the case of PYS from solutions, nuclear reorganisation energies are more important than in the solid state since molecules are far less constrained. The Franck-Condon principle in the context of photoemission assumes that a molecule undergoing photoionization experiences no significant change in the positions of the nuclei of the emitter relative to its environment. After photoemission, the system undergoes reorganisation, giving rise to changes in the nuclear coordinates of the emitter, λ_1_, and also relative to its wider environment, λ_2_. In solution, the reorganisation of the solvent molecules gives rise to a negative free energy term, thereby lowering the transition energy. λ_1_ is accepted to be small for Fc^+^/Fc, on the order of ∼15 meV, due to very minor variations of the molecular geometry upon oxidation and reduction.^38^ Solvent reorganisation, λ_2_, is somewhat larger, on the order of 780 meV.^38^ The adiabatic ionization potential is the minimum energy required to remove an electron from the molecule, and so therefore observed first in a PYS experiment, whereas the vertical transition requires no reorganisation and so has a higher energy. In a vertical photoemission event, however, photoemission is largely independent of slow solvent reorganisation and so the transition is strongly selected for and results in a more pronounced photocurrent.^62^ In general, the redox potential recovered in CV is equivalent to the adiabatic photoemission onset, although when completely irreversible waves are measured by CV, an equilibrium potential is not present, and the recovered value will be dependent on local experimental conditions.^62^

We used the ambient pressure photoemission method to determine the adiabatic IE of 51 mM ferrocene in acetonitrile to be −4.94 ± 0.05 eV. The photoemission spectrum from the acetonitrile solvent alone has no measurable photoelectron response between 3.6 and 7 eV (see SI Section 4). The concentration of ferrocene in solution was varied between 0.05 and 51 mM and the photoemission yield was measured (see Figure 2D). As expected, the photoemission yield above threshold increases with ferrocene concentration, confirming that the measured signal originates from ionisation of ferrocene molecules. For concentrations where electrons could be detected above the noise level, changes in threshold values were observed with increasing concentration, with IE decreasing from −5.02 ± 0.05 eV for 43 mM, to -5.00 ± 0.05 eV for 30 mM and -5.05 ± 0.05 eV for 14 mM, -5.07 ± 0.05 eV for 6 mM, -5.14 ± 0.07 eV for 2.5 mM and -5.42 ± 0.1 eV for 0.05 mM (see Figure 2F). Concentration dependence in IEs of this form has been observed for many solutes and solvents used in PYS (see discussion in SI Section 2).^27–29,31,63,64^ Our confidence intervals of the fit for all measurements apart from the two lowest concentrations (see SI Section 5) are smaller than our experimental uncertainty arising from spectral dispersion of the input beam.

To explain the change of IE with concentration, we note the increase of the best fit *n* in eq 2 as a function of increasing concentration (see Figure 2E). Our results are consistent with a weak image force at high concentrations where *n* ≈ 5/2 to a stronger barrier at low concentrations (*n* ≈ 2). We therefore suggest that high concentrations act to alter the nature of the surface potential and screen the image force. The physical origin of the screening force is beyond the scope of this work, although we discuss theoretical and experimental evidence of screening mechanisms in SI Section 2. As the image force diminishes at high concentrations, the 51 mM solution, near saturation at room temperature, not only offers the best signal-to-noise ratio but also the best fit to the domain associated with minimal influence of the image force.

We now compare our measured −4.94 ± 0.05 eV value to other literature measurements of the Fc^+^/Fc couple. Maya^65^ et al. compare gas phase UPS of Fc to PYS in solution of Fc, determining -6.90 and -5.83 eV, respectively. The 6.90 eV of emission of solid ferrocene into the gas phase is consistent with other reports^66–70^ (see SI Section 1). Maya et al. concluded that the -5.83 eV value from PYS in solution referred to the vertical ionisation energy, and the solvent polarisation effect was responsible for the ∼1.12 eV shift.^65^ Solvent reorganisation energy λ_2_ of Fc^+^/Fc in acetonitrile is predicted to be on the order of ∼0.8 eV,^38^ which would suggest an adiabatic photoemission level of ∼-5.03 eV, a value that is broadly consistent with our report. We assume for reasons of sensitivity and temperature that only the intense vertical effect was observed in the study by Maya et al. As ferrocene is known to rapidly degrade under X-ray irradiation,^71^ we suppose for this reason there are no reports of high-energy ferrocene liquid-jet measurements. Recent computational work by Makos et al. utilising a dynamic electron and solvent relaxation model with fragment potentials determined the energy of the Fc^+^/Fc couple as -4.94 eV.^55^ We believe the -4.94 eV^55^ value is consistent with our result.^37,55^ We note our results are not consistent with other widely reported reference values, which calls for a revaluation of some electrochemical references and the corresponding extrapolated HOMO/LUMO values reported for many organic semiconductor materials (see Figure 1 and SI Section 1).

In conclusion, we attempt to resolve a long-standing ambiguity that has persisted in mapping electrochemical values to the vacuum scale using an absolute measurement approach in solution. Our measurements give significant confidence to the -4.93 eV for Fc^+^/Fc extant literature value, as we find a corresponding value of −4.94 ± 0.05 eV from absolute photoemission measurements. We find other values inconsistent with our observations. A trivial extension of our reported method to other analytes will allow for the absolute determination of the energy levels of organic semiconductor compounds in solution in ambient conditions. Usefully, the electrochemical stability window of a particular solvent has no bearing on the measurement and there is no requirement for degassing or conducting the measurement under vacuum. We believe that future measurements will aid a mechanistic understanding to the relatively unexplained ∼0.3 eV error in relating solution measurements to the solid-state fil devices.^15^ It is also a significant conclusion that in high-sensitivity PYS measurements that the adiabatic ionisation energy may be observed.

## Methods

### Ambient Photoemission Yield Spectroscopy

Samples were prepared for photoemission by injecting the solution into a metal sample holder, which constituted of a disk with a machined dimple. The sample holder exhibited no photoemission up to 7.5 eV illumination (see control measurements in SI Section 5). A solution of ferrocene in acetonitrile was made and then syringed onto the dimple, until the liquid formed a meniscus. An adapted Kelvin probe, from the APS02, KP Technology, described elsewhere, was utilised to measure photocurrent. APS measurements were taken using the Photoemission System (APS020) (KP Technology). The nitrogen flow was turned on 30 min prior to measurements to prevent the formation of ozone from the UV bulb. Once oxygen levels were sufficiently low, the UV bulb was turned on and allowed to stabilize for 10 min. Electrical sample ground was confirmed. The Kelvin probe was enabled, the probe slowly moved toward the sample surface. LED light was used to direct the positioning of the UV beam. The averaging and gain were set to optimise the measurement. Care was taken to operate quickly to minimize potential oxygenic degradation of the Fc/Fc^+^ sample. Paul et al. have measured the degradation of modified Fc complexes to be on the order of 10 h and consider it robust up to 400 °C (we operate at room temperature).^38^ Most measurements took approximately 90 seconds. To find the threshold energy for photoemission, the dark background was first subtracted from the raw data before a spectral calibration accounting for the wavelength-dependent intensity of the lamp was applied.

### Absorption Spectra

Absorption spectra were taken using a Varian CARY 300 absorption spectrometer in transmission mode. Solutions to be measured were placed in a Cole-Parmer 10 mm path length quartz cuvette, and the transmission of light through the solutions was compared to the transmission of an empty cuvette to obtain an absorbance value.

### Cyclic Voltammetry

An electrochemical Analyzer potentiostat model 620E from CH Instruments was used for Cyclic Voltammetry (CV) analysis. Solutions of fac-Tris­(1-phenyl-3-methylimidazolin-2-ylidene)-C,C(2)’iridium­(III) in MeCN were prepared and bubbled with MeCN saturated nitrogen gas for 15 min before measurements. 0.1 M MeCN solution of TBAPF6 was used as electrolyte solution. The working electrode was glassy carbon, reference electrode aqueous Ag/AgCl and the counter electrode Pt wire. The redox potentials are reported relative to a saturated calomel electrode (SCE) with a ferrocene/ferrocenium (Fc/Fc^+^) redox couple (−4.94 eV) as the internal standard.

## Supplementary Material



## Data Availability

The research data and code supporting this publication^73^ can be accessed at 10.17630/8d3989f0-38c1-4b9e-84cd-30859be7476c and at https://github.com/Tb8854/Ferrocene_PYS_Paper.

## Footnotes

1 3rd Edition refers to −4.4 eV for NHE, −4.6 eV for the SCE (0.244 V between SCE and NHE), 2nd edition to −4.5 eV for NHE and −4.7 eV for SCE (0.242 V between NHE and SCE) and the first edition 0.242 V between NHE and SCE. 1st, 2nd and 3rd editions give 0.77 V for E0 (Fe^3+^/Fe^2+^) vs NHE and 0.53 V for E0 (Fe^3+^/Fe^2+^) vs SCE.

## Notes

Various reportedliterature comparisons of electrode scaling and relevant UPS measurements; CV comparison table; thermodynamics of surface potential; control measurements; parameter fits (PDF)
