# Supplementary material for: Absolute Calibration for Cyclic Voltammetry from the Solution-Phase Ionisation of Ferrocene
Source: ACS Electrochem. 2026 Jan 20;2(2):297–304. doi: 10.1021/acselectrochem.5c00382 (PMC12884449; doi:10.1021/acselectrochem.5c00382)
Supplement: Supplementary file 1 [file ec5c00382_si_001.pdf]

SUPPLEMENTARY INFORMATION

For

Absolute calibration for cyclic voltammetry from the solution-phase ionisation of ferrocene

Tomi K. Baikie[1,2,3], Jonathon R. Harwell[1], Iain D. Baikie[1,4], Eli Zysman-Colman*[5], Ifor D. W. Samuel*[1] and Graham A. Turnbull*[1]

[1]Organic Semiconductor Centre, SUPA, School of Physics and Astronomy, University of St Andrews, St Andrews, KY16 9SS, UK.

[2]Cavendish Laboratory, University of Cambridge, Cambridge CB3 0EH, UK

[3]Research Laboratory of Electronics, Massachusetts Institute of Technology, Cambridge, Massachusetts, USA, 02139

[4]KP Technology Ltd, The Old Foundry, Burn Street, Wick, KW1 5LE, UK

[5]Organic Semiconductor Centre, EaStCHEM School of Chemistry, University of St Andrews, St Andrews, KY16 9ST, UK

Corresponding Authors:

Eli Zysman-Colman - eli.zysman-colman@st-andrews.ac.uk

Ifor D.W. Samuel - idws@st-andrews.ac.uk

Graham A. Turnbull - gat@st-andrews.ac.uk

Table of Contents

SI Section 1- CV Comparison Table **2**

SI Section 2 – Introduction to Theory **4**

SI Section 3 – Thermodynamics of Surface Potential **13**

SI Section 4 – Parameter Fits **14**

SI Section 5 – Control Measurements **15**

SI Section 1- CV Comparison Table

Table SI 1 outlines various literature comparisons of electrochemical measurement scaling as a function of electrode and reference, or relevant UPS measurements, we have highlighted in blue the values that are supported by our result. We refer the reader to excellent reviews on the (lack of) standardization of the reference electrodes and the impact this has on the Fc^{+}/Fc couple, particularly those by Pavlishchuk *et al.* [1] and Cardona *et al.* [2], from which much of **Table SI 1** is derived.

Further, when determining the HOMO/LUMO of organic compounds,[3] perhaps as a matter of convention within a community, some identify the threshold value by either selecting their onset, halfway up the onset, or even the peak to define the band[4]. We believe it is pertinent in charge transport applications that the onset of the band defines its electrical properties. While not a limitation of technique, but rather one of convention, there is an inconvenient discrepancy when comparing values from UPS directly to PYS and CV, since UPS typically uses the peak whereas CV variously defines the onset or sometimes (E_{pa}+E_{pc})/2. Usefully, PYS values from solution can be easily recovered from UPS measurements using a simple sum when both measure the same band[5]. Without doing this, depending on the nature of the band, the comparative difference in PYS and UPS can be on the order of ~0.5 - 1 eV for molecular species.[4,6]

We here briefly comment on the nomenclature of the HOMO level in relation to DFT studies. The ionisation energy is the energy required to remove an electron to “just beyond” the subject material. To repeat, the ionisation energy is the energy cost of removing that electron, including all relaxation and secondary interactions. The HOMO level is often used to refer to the same concept, the ionisation energy in molecular species. However, it is also used to reflect the 2-electron-containing occupied molecular orbital of the highest energy in the ground state. This term, therefore, does not always account for the relaxation and secondary interactions of removing an electron from this level. In quantum chemistry the definition can become nebulous depending on the context, but the HOMO is often related by Koopman’s theorem or Janak’s theorem to the ionisation energy. The exact approximations differ for different methodologies, but very broadly, the picture is that when an electron is removed, the orbitals remain frozen, and the second-order interactions are not always well accounted for. This gives rise to secondary correction terms, for example a “post-DFT” method that might account

for electron interactions and relaxation. This is particularly true in small molecules where the electron may be strongly localised since removing an electron can radically reshape the electron density and so a stationary HOMO is likely a poor proxy. We refer to the literature for further discussion.[7]

Table S1. Various reported literature comparisons of electrode scaling and relevant UPS measurements.

REFERENCE	VALUE	REFERENCE	VALUE
FC/FC⁺ VS SCE IN MECN	0.38 V [1]	0V NHE vs Fermi Scale	-4.5 eV[8]
FC/FC⁺ VS SCE IN MECN	0.40 V [9]	0V NHE vs Fermi Scale	-4.456 eV[2]
FC/FC⁺ VS SCE IN MECN	0.41 V [10]	0V NHE vs Fermi Scale	-4.4 eV[11]
FC/FC⁺ VS SHE IN MECN	0.624 V[1]	0V NHE vs Fermi Scale	-4.456 eV [12]
FC/FC⁺ VS SHE IN MECN	0.028 V[1]	0V NHE vs Fermi Scale	-4.75 eV[13]
FC/FC⁺ VS SHE IN MECN	0.403 V[14]	0V NHE vs Fermi Scale	-4.7 eV[15]
FC/FC⁺ VS SCE VS FERMI	-4.60 V[16]	0V NHE vs Fermi Scale	-4. 73 eV[17]
FC/FC⁺ VS ?? IN MECN	-4.644 V[18]	0V NHE vs Fermi Scale	-4.85 eV[19]
FC/FC⁺ VS ?? IN MECN	-4.964[20]	SCE vs NHE	0.24 V [8]
FC/FC⁺ VS FERMI SCALE	-5.1 eV[21]	SCE vs NHE	0.25 V [1]
FC/FC⁺ VS FERMI SCALE	-4.75 eV [21]	0 V SCE vs Fermi Scale	-4.456 eV
FC/FC⁺ VS FERMI SCALE	-4.85 eV	0 V SCE vs Fermi Scale	-4.4 eV[21,22]
FC/FC⁺ VS FERMI SCALE	-5.39 eV[21]	0 V SCE vs Fermi Scale	-4.4 eV[21,22]
FC/FC⁺ VS FERMI SCALE	-4.8 eV [23] [2421]	SCE water vs SCE Acetonitrile	-0.141[18]
FC/FC⁺ VS FERMI SCALE	-4.928 eV[25]	SHE water	4.281 V[26]
FC/FC⁺ VS FERMI SCALE	-4.94 eV[25]	SCE water	4.522 V[26]
FC/FC⁺ VS FERMI SCALE	-4.6 eV[21]	SCE Acetonitrile	4.188V[27,28]
FC/FC⁺ VS FERMI SCALE	-4.98[20]	SHE	3.83 – 4.66 V[29,30]
FC/FC⁺ VS FERMI SCALE	-4.644 [18]	SHE	4.36 or 4.43 [30,31] [32]
FC/FC⁺ GAS PHASE	-6.92 eV[33]	SCE in MeCN	4.6[20]
FC/FC⁺ GAS PHASE	-6.91 eV[34]	SHE in MeCN	4.6[35]
FC/FC⁺ GAS PHASE	-6.90 eV[36]	SCE in MeCN	4.381[18]

FC/FC+ GAS PHASE	-6.85 eV [36]	SCE in MeCN	4.381[18]
FC/FC+ GAS PHASE	-7.062 eV[20]		
FC/FC+ GAS PHASE	-7.047 eV [20]		
FC/FC+ GAS PHASE	-6.747 eV[18]		
FC/FC+ GAS PHASE	-6.9 eV [37,38]		
FC/FC+ GAS PHASE	6.99[39]		
FC/FC+ GAS PHASE	6.81[40]		
FC/FC+ GAS PHASE	6.82[41]		
FC/FC+ GAS PHASE	6.72[42]		
FC/FC+ GAS PHASE	6.97[36]		
FC/FC+ GAS PHASE	6.87[18,43]		
FC/FC+ GAS PHASE	6.763[18]		
FC/FC+ GAS PHASE	6.783 [18]		
FC/FC+ GAS PHASE	6.790[18]		

SI Section 2 –Theory Approach

We here outline a brief introduction to our approach in determining photoelectron current near the onset of emission from solution. Pope described emission from solution into the gas phase in 1962[44] and the foundations for theoretical considerations of photoemission from solutions were later laid by Delahay[45], making an early phenomenological observation that the measured photocurrent could be described by a power law. Emission spectra have thereafter been fitted to empirical power laws [5,6,45–49]. The Brodsky (sometimes Brodskii) theory of photoelectron emission by solutions is of great relevance to liquid interfaces and our approach is largely based on the contributions by Brodskii [50–52] and Gurevitch[47,53].

Electrons in polar media, such as the aqueous electrolyte solutions commonly used in cyclic voltammetry, are termed “localised” when their contribution to the current is by hopping/diffusion or “delocalized” when they contribute directly to the current. As opposed to the situation in vacuum, where free electrons can exist indefinitely, photoelectrons emitted into a solution thermalize and eventually become solvated (or localised). The formation of delocalized electrons is not only possible as a result of direct photoionization, but also possible from radiation treatments or

chemical processes [54]. The photoelectrons therefore exhibit a band structure, with a relatively well-defined energy minimum that corresponds to the most stable configuration of the solvated electron, analogous to a localized state in a disordered system[55], but in the solution the disorder is provided by the fluctuating structure of the liquid. Analogously, the delocalised electrons may be described by a conduction band.

PYS in solution in our case can be broadly considered as a sequence of processes following:

1. Optical excitation of the emitting material.
2. Photoemission itself, following the Frank-Condon principle, where the solvent molecules retain the same orientation around the emitter as they had before the escape of the electron.
3. Reorganisation of the solvent and emitter, with a cost of returning the solution to equilibrium known as the reorganization energy.

Then the electron may either undergo:

4. Thermalization and solvation of emitted electrons, resulting in a decrease of their initial energy to the mean energy of thermal movement and formation of a localised electron.

Or,

5. Propagation of delocalized electrons whereupon some can escape from the solution into the vapour phase, overcoming any image force or potential barrier at the interface.[47]

Electrons are emitted into the gas phase from a layer of solution having a thickness of the order of the thermalization length of low-energy electrons, on the order of $\sim 2-4$ nm. Consequently, there is little attenuation of the photon flux as a result of absorption of the solvent from which emitted electrons originate[45]. The dependence of the photocurrent on $h\nu$ is considered here to be largely dependent on step 1 & 5, as steps 2 through 4 are largely independent of electron energy near the ionisation onset.

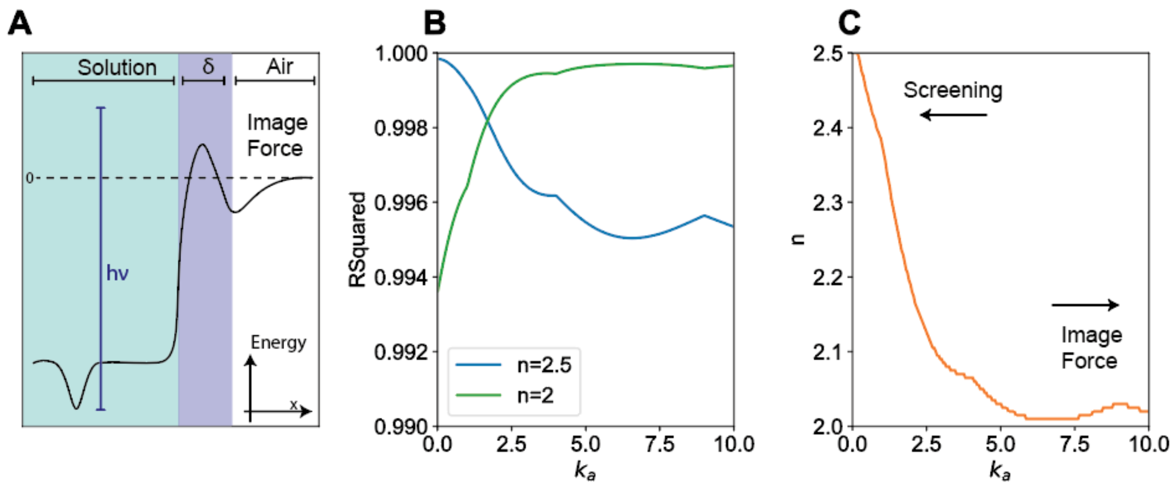

Figure S1. A – Potential and energy parameters of model. **B** – Quality of fit for photocurrent when treated with either $n = 2.5$ or $n = 2$ as a function of the Coulomb momentum k_α **C** – Plot of the value of n that gives optimised fit as a function of k_α highlighting the transition between Coulomb-like image force at high k_α to complete screening at $k_\alpha = 0$.

We treat the transport of electrons as the propagation of spherical waves centred on emitting molecules. Much of the approach is similar to that of Sommerfeld's spherical wave propagation in a semi-infinite medium[56]. The interaction with the medium is accounted for by an exponential attenuation factor, λ. λ is a damping term that accounts for various loss mechanisms near the energy threshold of emission such as inelastic scattering, generation of phonons by electrons, Landau damping, and elastic scattering with changes in the direction whereupon the electrons can no longer overcome the energy barrier at the surface. A cartoon schematic of the problem is given in **Figure S1**.

Supposing that the delocalised photoelectron in solution is described by ψ. Transmission through the interfacial barrier corresponds to the transmitted wave, ψ_t, so

$$\psi = \begin{cases} \psi_s + \psi_r & \text{for } x < 0 \\ \psi_t & \text{for } x > \delta > 0 \end{cases}$$

Equation SI 1

where ψ_s is the source wave, ψ_r is the reflected wave and ψ_t is the wave that has crossed the interface and δ is a region near the surface, which we address in detail later. The emitting function ψ_s satisfies the Schrödinger equation with the potential $V(x)$,

$$V(x) = \begin{cases} v(x) \text{ for } x > \delta \\ \text{Exact Form Unknown for } 0 < x < \delta \\ -U - i\dfrac{\hbar}{\mu_s \lambda}\sqrt{2\mu_s(E+U)} \text{ for } x < 0 \end{cases}$$

Equation SI 2

where x is the component of the spherical wave towards the liquid surface and E and U, have their usual meanings, kinetic energy and potential energy, respectively. ψ_s is constructed from spherical harmonics,

$$\psi_s \propto i\, \mu_s\, A_{l,m}\, Y(l,m)\, R(r)$$

Equation SI 3

where μ_s is the electron mass in solution, $Y(l,m)$ is the spherical harmonic, $R(r)$ gives a radial dependence and $|A|^2$ corresponds to the photon absorption cross section. With many emitters, **Equation SI 3** corresponds to the n-photoelectron solution and represents a superposition of spherical waves emerging with different angular momenta l and projections of angular momenta m along the x axis, $A_{l,m}$ is associated with the corresponding absorption cross sections.

A solution to ψ_r and ψ_t can then be obtained by defining a transfer coefficient, B, and assuming continuity. Importantly, continuity can be assumed outside the boundary region δ which eliminates the influence of the narrow boundary region, δ, where the exact form of the potential is not known and likely complicated. The transmitted wavefunction as $x \to \infty$ can then be written as

$$\psi_t \propto \int B\, \psi_s(\boldsymbol{k}_\parallel)\, e^{i\, \boldsymbol{k}_\parallel\, \boldsymbol{x}}\, d\boldsymbol{k}_\parallel.$$

Equation SI 4

The form of B can be derived through the linear combination of input and output waves, and here we assume a form $B \propto \left(\sqrt{k_x^f}\right)^{-1}$ that is consistent with the absence of a long-range Coulomb potential[50,52], where k_x^f is the final momentum in the emission direction of the electron. We note here that there exists discrepancy of the form of B between Brodskii's early and later works (ref [52]

and [50]), and we pick B such that it is consistent with Gurevitch[47] and Benderskii[57]. We also note that at higher emitter concentration the form of B should allow for a possible influence of a coherent superposition of emitted waves which may account for a nonlinear dependence of photoemission on concentration of emitting centres[50,58]. In any case, we soon relax our assumption on the exact form of B using results from scattering theory.

An expression for the corresponding photocurrent can be determined by using properties of spherical harmonic orthogonality and can be made particularly clear by taking the Fourier transform of **Equation SI 4** (for exact form see Equation 15 in Ref[52]). The photocurrent j as $x \to \infty$ is given by

$$\begin{aligned} \boldsymbol{j}(E_f, x) \quad &\propto \int \left(\psi_\mathrm{t} \frac{\partial \psi_\mathrm{t}^*}{\partial x} - \psi_\mathrm{t}^* \frac{\partial \psi_\mathrm{t}}{\partial x}\right) \mathrm{d}y\, \mathrm{d}z \\ &\propto \int |B|^2\, \Theta\left(\left(k_x^f\right)^2\right) \boldsymbol{k}^f \left|\tilde{\psi}_s(k_\parallel)\right|^2 e^{\beta(\lambda) x_0}\, \mathrm{d}^2 k_\parallel \end{aligned}$$

Equation SI 5

where Θ is the Heaviside step function, $\boldsymbol{k}^f = \left(k_\parallel, k_x^f\right)$ and $e^{\beta(\lambda)x_0}$ where β is a function of λ. We now compute the flux of $\boldsymbol{j}$ over the surface of the solution defined by $\boldsymbol{n} = \left(\sin\theta\cos\phi\cos\alpha_p, \sin\theta\sin\phi\sin\alpha_p, \cos\theta\right)$. Here (θ, ϕ) defines the observation direction and $\alpha_p$is polarisation angle (we here assume circularly polarised light), hence

$$\boldsymbol{j} \cdot \boldsymbol{n} \propto \int |B|^2 |A|^2\, \Theta\left(\left(k_x^f\right)^2\right) \left(\boldsymbol{k}^f \cdot \boldsymbol{n}\right) \left|\tilde{\psi}_s(k_\parallel)\right|^2 e^{\beta(\lambda) x_0}\, \mathrm{d}^2 k_\parallel$$

$$\begin{aligned} &\propto \int_0^\infty \int_0^{2\pi} |B|^2 |A|^2 k_\parallel\, \Theta\left(\left(k_x^f\right)^2\right) \left(k_x^f \cos\theta + k_\parallel \sin\theta \left(\sin\phi\sin\alpha_p + \cos\phi\cos\alpha_p\right)\right) \left|\tilde{\psi}_s(k_\parallel)\right|^2 d\alpha_p dk_\parallel \\ &\propto \int_0^\infty \int_0^{2\pi} |B|^2 |A|^2 k_\parallel \Theta\left(\left(k_x^f\right)^2\right) \left(k_x^f \cos\theta + k_\parallel \sin\theta \left(\cos(\phi - \alpha_p)\right)\right) \left|\tilde{\psi}_s(k_\parallel)\right|^2 d\alpha_p dk_\parallel \end{aligned}$$

Equation SI 6

Expressing $\left(p_3^f\right)^2 = 2m_f E_f - p_\parallel^2$ transforms

$$\int_0^\infty p_\parallel \Theta\left(p_3^f\right) dp_\parallel = \int_0^\infty p_\parallel \Theta\left(\sqrt{2m_f E_f - p_\parallel^2}\right) dp_\parallel$$

$$= \Theta(E_f) \int_0^{\sqrt{(2m_f E_f)}} p_{\parallel} dp_{\parallel},$$

Equation SI 7

where the Heaviside function forces the integrand function to zero when $p_{\parallel}^2 \geq 2m_f E_f$, and defining $E_f > 0$ we can now drop the Heaviside function entire and change the integration limits in **Equation SI 6**,

$$(\mathbf{j} \cdot \mathbf{n}) \propto \int_0^{\sqrt{(2m_f E_f)}} \int_0^{2\pi} |B|^2 |A|^2 \, p_{\parallel} \big(p_3^f \cos\theta + p_{\parallel} \sin\theta \cos(\phi - \alpha_p)\big) \big|\tilde{\psi}_s(p_{\parallel})\big|^2 d\alpha_p \, dp_{\parallel}.$$

Equation SI 8

The only contributions to $\big|\tilde{\psi}_s(p_{\parallel})\big|^2$ come from $\big|A_{l,m}\big|^2$ and can be determined from the one-particle dipole approximation and in our case will be dominated by $l = 1, m = 0$ (see **Figure 2** in the main text), which is approximately constant, hence the current above the sample follows,

$$(\mathbf{j} \cdot \mathbf{n}) \propto \int_0^{\sqrt{(2m_f E_f)}} \int_0^{2\pi} \big(p_3^f \cos\theta + p_{\parallel} \sin\theta \cos(\phi - \alpha_p)\big) d\alpha_p \, dp_{\parallel}$$

$$\propto \int_0^{\sqrt{(2m_f E_f)}} \int_0^{2\pi} \left(\left(2\mathrm{m_f} E_f - p_{\parallel}^2\right)^{\frac{1}{2}} \cos\theta + p_{\parallel} \sin\theta \cos(\phi - \alpha_p) \right) d\alpha_p \, dp_{\parallel}$$

which may be solved (in your favourite computer algebra system) to find that,

$$\propto E_f = h(\nu - \nu_0)$$

Equation SI 9

The Brodskii photocurrent is then $(\mathbf{j} \cdot \mathbf{n})$ times the density of states, which Brodskii assumed to increase as a first order power approximation as $\sqrt{E_f}$,

$$I \propto \int_0^{E_i + h\nu} (\mathbf{j} \cdot \mathbf{n}) \; \rho(E_f) \, dE_f = \int_0^{E_i + h\nu} E_f \sqrt{E_f} \, dE_f \propto (\nu - \nu_0)^{\frac{5}{2}}$$

Equation SI 10

It is also possible to approach the solution using scattering theory, an approach first pioneered by Gurevich[47,51,53] and here adapted for solutions by Benderskii and Grebenshchikov[57]. Using the Jost solution[59,60] of the radial Schrodinger equation in electron propagation, an expression that accounts for varying λ and B can be found. This approach, though more complex, has the advantage that a continuous expression between screening of the image force and presence of the image force can be found, consistent with the results in **Figure 2E**. The current is given by Equation 8 in ref[57] , reproduced here,

$$j(k) = \frac{k_\alpha \sinh(2\pi Lk)}{\cosh(2\pi Lk) - \cosh\left\{2\pi Lk\left(1-\left(\frac{k_\alpha}{Lk^2}\right)\right)^{\frac{1}{2}}\right\}},$$

Equation SI 11

where L is the scattering length and $k_\alpha \propto U_0/\epsilon$ is the Coulomb momentum that describes the long-range Coulomb interaction U_0 and where ϵ is the dielectric permittivity. The total photocurrent is given similarly to that of **Equation SI 10**,

$$I \propto \int_0^{E_i+h\nu} j\left(\sqrt{2mE_f}\right)\ \rho\left(E_f\right)\, dE_f = \int_0^{E_i+h\nu} j\left(\sqrt{2mE_f}\right) \ln\left\{1 + exp\left(\frac{\hbar\omega + \mu - E_f}{k_B T}\right)\right\}\, dE_f.$$

Equation SI 12

Equation SI 12 can be numerically solved. We kept L fixed at 1 nm, since electrons emitted into the gas phase originate between 2 to 4 nm[45] from the surface. **Figure S1B** plots the quality of fit of **Equation SI 12** after a $I^{1/n}$ treatment, where $n = \{2.5, 2\}$, with a clear transition observed between $n = 2$ fitting the best and $n = 2.5$ as k_α changes. **Figure S1C** plots an optimised $n \in \mathbb{R}$ for R squared linearity versus k_α, where a continuous transition between $n = 2$ and 2.5 is observed. Large values of k_α correspond to a long-range Coulomb force giving a form similar to that of the well-known Fowler law, to lack of any long-range force at small k_α which corresponds to complete screening and the $5/2$ Brodskii law.

To illustrate the above, the schematic in **Figure 2A-C** in the main text plots the spherical harmonics $|Y_l^m(\theta,\phi)|^2$ scaled by λ as a function of depth which act as an initial condition for **Equation SI 13**,

$$\frac{d^2\psi}{dt^2} - \nabla^2_{x,y,z}\psi = \mathrm{NeumannValue}\left[-\frac{d\psi}{dt}\right],$$

Equation SI 13

where the appropriate Neumann values and Dirichlet conditions are used to model varying portions of reflection or transmission at the appropriate boundaries.

Experimental reports consistently find the 5/2 Brodskii law[45,47,49,51,53,54,57,61–63] and concentration dependence on either n and the IE[6,58,64–69]. We caution that the modelling approach we follow through **Equation SIs 1 – 12** is effectively empirical, as we enforce a change in the image potential from Coulomb like $(n = 2)$ in the metal case to a completely screened potential $\left(n = \frac{5}{2}\right)$ with little physical justification. The physical origin underpinning the mechanism of image force screening remains unclear to us. Brodskii suggests dielectric screening, yet the dielectric constant required for screening in the $5/2$ power law cannot be attained in most solvents [47]. We here claim that the necessity of the absence of image forces required for **Equation SI 11** in deriving the $5/2$ law in solution remains an unproven postulate and we suspect that the answer lies in a full description of multiple electron scattering in dense media, which remains unsolved.[70–72]

Nevertheless, we can attempt to give a back-of-the-envelope sanity check on the reduction of the potential from the image force, or more broadly, the total potential felt by a photoelectron in solution escaping solution. **Equation SI 11** enforces the transition from Coulomb like (when $k_\alpha/k \gg 1$) to fully screened for ($k_\alpha/k \ll 1$), as such we associate $k_\alpha = \pi a_\alpha^{-1}$ with a Debye depth of the image force, a_α. Therefore for $L/a_\alpha \approx 2$ we expect a fully screened potential so $n \to 5/2$, then an intermediate region for $L/a_\alpha \approx$ 5-7, and a Coulomb limit for larger values where $n \to 2$. Supposing that we fix $a_0 \approx 0.2$ nm, consistent with the free electron mean free path of water[73], this suggests a change in the scattering length from $L = 0.4$ nm at high concentrations to $L > 1$ nm at low concentrations. Although a decrease in scattering length with increasing concentration follows an intuitive model, we note that change scattering length with concentration have been previously

observed in similar systems[74–76] and exhibit complex behaviour, as does the relative increase in solute density solute molecules near the surface[77,78]. Conversely, if we fix the characteristic scattering length scale, $L \approx 1$ nm, we have a range of $a_\alpha = 0.5$ nm at high concentrations to $a_\alpha < 0.2$ nm at low concentrations, meaning the Debye length increases with increasing concentration. This picture is as a first-order approximation consistent with a stronger screening due to increased ionic strength resulting in a shorter Debye length. We note that order of magnitude changes in a_α have been observed for increasing concentration of solute molecules[79–81]. We believe a combination of changes in L and a_α as a function of concentration are likely to underpin the change in the effective image force felt by a photoelectron, which in turn alters the nature of n.

In the preceding section we have further assumed that the potential in the δ region is not changing, yet the magnitude and form of the interface potential is almost certainty changing as a function of concentration, which would give rise to a change in IE as a function of concentration. We suppose that acetonitrile, as previously reported, organises as a bilayer[82,83] at the liquid/gas interface at low concentrations of ferrocene, as the concentration increases, this organisation is disrupted by an increase in concentration of the solute. This directly impacts magnitude of the surface and location of the surface barrier as evidenced in molecular dynamics simulations[82] and evidenced by experiments probing the effective surface potential by up to ~0.1V. [82,83]

We reiterate again that we take no strong view on the origin of changes in the surface barrier as a function of concentration but confirm again that it is easily measured at a function of the n between two concentration limits; Coulomb (metal)-like ($n = 2$) at high concentrations and complete image force screening-like ($n = 5/2$) at low concentrations.

SI Section 3 – Thermodynamics of Surface Potential

The photoionization energy in solutions is variously defined throughout the literature. We define the photoionization energy, for an electron to be emitted *into the solution* to be,

$$\phi_{\mathrm{solution}\rightarrow\mathrm{solution}^*} \;:\; \mathrm{X} \rightarrow \mathrm{X}^{*+} + \mathrm{e}^-$$

Equation SI 14

which defines the ionisation energy dependent on moving a localised electron to a delocalised one in solution. X^{*+} refers to the initial state given by the Frank-Condon principle, before nuclear reorganisation takes place. $\phi_{\mathrm{solution}\rightarrow\mathrm{solution}^*}$ is sometimes referred to as the nonequilibrium or vertical work function for a solution. The free energy for **Equation SI 14** is known as the free energy of emission ΔG_e.

Dipole-solvent interactions cause the minimum energies required for photoionization to vary as both nuclear and electronic contributions giving rise to an energetic shift compared to the ionisation energy in vacuum, ϕ, so

$$\phi_{\mathrm{solution}\rightarrow\mathrm{solution}} = \phi + \text{nuclear reorganisation} + \text{electronic dispersion}$$

Equation SI 15

Nuclear reorganisation and electronic dispersion are generally negligible in gas-phase photoionization as the prevailing distances between adjacent molecules are too great to allow significant transition dipole-molecule interactions. In the main text we describe $\phi_{\mathrm{solution}\rightarrow\mathrm{gas}}$, which corresponds to

$$\phi_{\mathrm{solution}\rightarrow\mathrm{gas}} \;:\; \mathrm{X}_{\mathrm{Solution}} \rightarrow \mathrm{X}^{*+}_{\mathrm{Solution}^*} + \mathrm{e}^-_{\mathrm{Solution}} \rightarrow \mathrm{X}^{+}_{\mathrm{Solution}} + \mathrm{e}^-_{\mathrm{Gas}}$$

Equation SI 16

Where $\mathrm{X}^{*+}_{\mathrm{Solution}}$ and $\mathrm{X}^{+}_{\mathrm{Solution}}$ corresponds to the excited and relaxed nuclear configuration, respectively. A negative free energy change corresponds to this relaxation, which we term the free energy of reorganisation ΔG_r, which in our case is the free energy change for the ion being inserted into a large volume of solution that carriers no net electrical charge. The adiabatic and vertical photoemission processes vary by ΔG_r. The total free energy change for $\phi_{\mathrm{solution}\rightarrow\mathrm{gas}}$ is then

$$\Delta G_{\Phi_{\mathrm{solution}\to\mathrm{gas}}} = \Delta G_e + \Delta G_r + |e|\chi,$$

Equation SI 17

where $|e|\chi$ is the electronic charge multiplied by the surface potential of the solution, χ. $|e|\chi$ is generally smaller than < 10 meV, see discussion[4,6,45,58,84–87]. Further, assuming that $\mathrm{X}^{+}_{\mathrm{Solution}}$ returns to a neutral state (which we suppose in our case), then the surface potential can be neglected almost entirely with the addition of positive charge, since $|e|\chi$ nearly cancels. [45]

SI Section 4 – Control Measurements

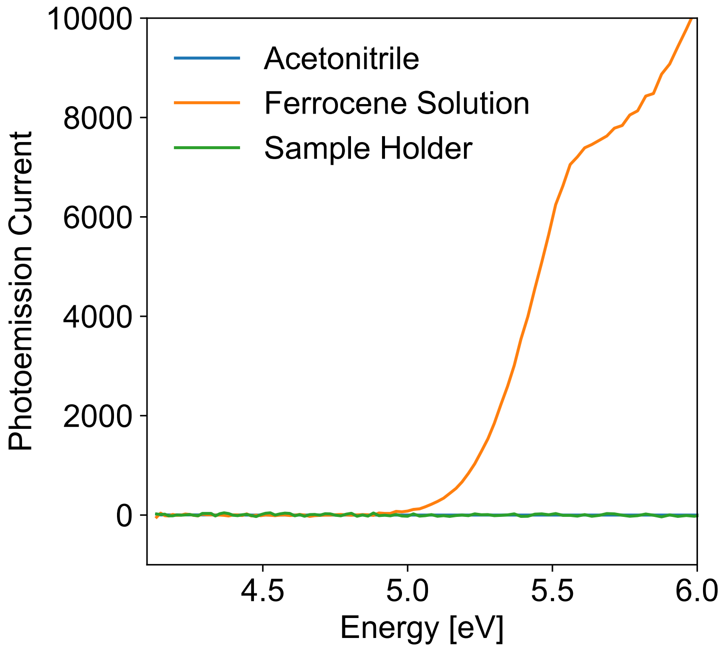

Figure S2 – Green line corresponds to the sample holder in Figure 1C in the main text. Blue line (which significantly overlaps with the green line) gives the response from just the solvent (acetonitrile) and orange line reproduces signal from Figure 2D in main text for 51 mM sample.

SI Section 5 – Parameter Fits

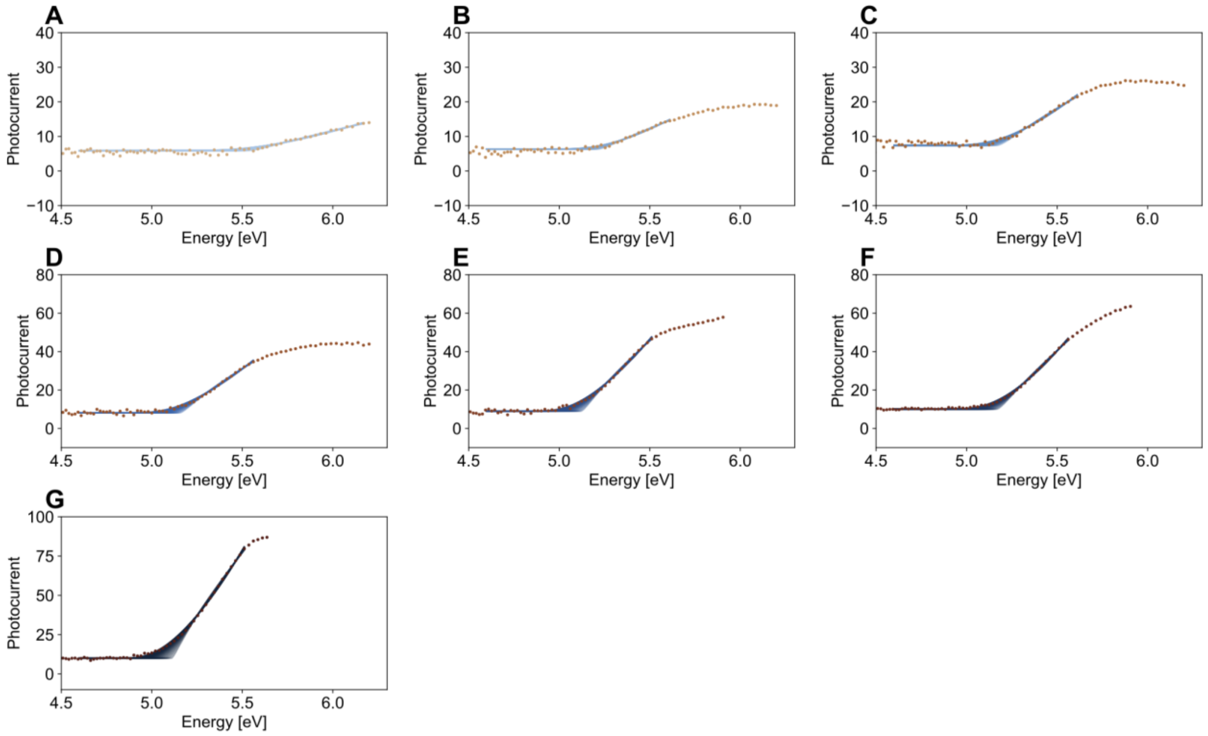

SI Figure 3 Photoemission yield spectra from ferrocene solutions in acetonitrile plotted after power law rule taken, highlighting linearity of fit. Measured photoelectron currents (dots) as a function of photon energy, with power law fits given. Increasing opacity refers to fits from $n = 1.5$ to 3.5 (quality of fit plotted in Figure 2E). Data has had an offset applied to account for discontinuity around 0. Fitting, code available at https://github.com/Tb8854/APS_Liquid, occurs in the linear space. **A** – 0.05 mM, **B** – 2.5 mM, **C** – 6mM, **D** – 14 mM, **E** – 30 mM, **F** – 43mM, **G** – 51 mM.

Table S2 outlines the best fit of n (3rd column) as defined in **Equation 2** in the main text used to determine ϕ. Column 4 gives the 95% confidence interval from the fit. The final column gives quality of fit measurements, specifically $\mathrm{AdjustedRSquared}$ accounts for the number of model parameters, AIC is the Akaike Information Criterion, BIC is the Bayesian Information Criterion, and RSquared is coefficient of determination R^2.

Table S2 – Parameter fits of **Equation 2** used to determine ϕ.

Concentration [mM]	ϕ *[eV]*	n	$\{AdjustedRSquared, AIC, BIC, RSquared\}$
51	-4.94 ± 0.05	2.6	$\{0.999, 402.279, 408.723, 0.999\}$
43	$-5.02 \pm$ 0.05 eV	2.5	$\{0.999, 344.01, 350.664, 0.999\}$

30	5.00 ± 0.05 eV	2.3	$\{0.999, 382.257, 388.7, 0.999\}$
14	5.05 ± 0.05	2.2	$\{0.998, 392.482, 399.137, 0.998\}$
6	5.07 ± 0.05	2.1	$\{0.996, 378.179, 385.034, 0.996\}$
2.5	5.14 ± 0.07	2	$\{0.993, 288.33, 294.066, 0.993\}$
0.05	5.42 ± 0.1	2	$\{0.991, 466.982, 474.549, 0.991\}$

References

(1) Pavlishchuk, V. V., & Addison, A. W. (2000). Conversion constants for redox potentials measured versus different reference electrodes in acetonitrile solutions at 25°C. Inorganica Chimica Acta, 298(1), . https://doi.org/10.1016/S0020-1693(99)00407-7

(2) Hansen, W. N.; Hansen, G. J. Absolute Half-Cell Potential: A Simple Direct Measurement. *Phys Rev A (Coll Park)* 1987, *36* (3), 1396–1402. https://doi.org/10.1103/PhysRevA.36.1396.

(3) Bredas, J.-L. Mind the Gap! *Mater. Horiz.* 2014. https://doi.org/10.1039/C3MH00098B.

(4) Seidel, R.; Faubel, M.; Winter, B.; Blumberger, J. Single-Ion Reorganization Free Energy of Aqueous Ru(Bpy)32+/3+ and Ru(H2O)62+/3+ from Photoemission Spectroscopy and Density Functional Molecular Dynamics Simulation. *J Am Chem Soc* 2009, *131* (44), 16127–16137. https://doi.org/10.1021/JA9047834.

(5) Maya, K.; Watanabe, I.; Ikeda, S. Photoelectron Emission Spectra of Butylamine, Ferrocene and Benzene Solutions Compared with Simulated Spectra Using Gas-Phase UPS. *J Electron Spectros Relat Phenomena* 1986, *40* (4), 307–315. https://doi.org/10.1016/0368-2048(86)80041-X.

(6) WatanabeIwao; MayaKeiichi; YabuharaYoshiki; IkedaShigero. Photoionization Threshold Energies of Amine Solutions Studied by Photoelectron Emission by Solution. 2006, *59* (3), 907–913. https://doi.org/10.1246/BCSJ.59.907.

(7) Zhang, G.; Musgrave, C. B. Comparison of DFT Methods for Molecular Orbital Eigenvalue Calculations. *Journal of Physical Chemistry A* 2007, *111* (8), 1554–1561. https://doi.org/10.1021/JP061633O/.

(8) Bard, A. J.; Faulkner, L. R. *Electrochemical Methods : Fundamentals and Applications*; Wiley, 2001.

(9) Connelly, N. G.; Geiger, W. E. Chemical Redox Agents for Organometallic Chemistry. *Chem Rev* 1996, *96* (2), 877–910. https://doi.org/10.1021/cr940053x.

(10) Cardona, C. M.; Li, W.; Kaifer, A. E.; Stockdale, D.; Bazan, G. C. Electrochemical Considerations for Determining Absolute Frontier Orbital Energy Levels of Conjugated Polymers for Solar Cell Applications. *Advanced Materials* 2011, *23* (20), 2367–2371. https://doi.org/10.1002/adma.201004554.

(11) Trasatti, S. The Absolute Electrode Potential: An Explanatory Note. *Pure and Applied Chemistry* 1986, *58* (7), 955–966. https://doi.org/10.1351/pac198658070955.

(12) Hansen, W. N.; Hansen, G. J. Absolute Half-Cell Potential: A Simple Direct Measurement. *Phys Rev A (Coll Park)* 1987, *36* (3), 1396–1402. https://doi.org/10.1103/PhysRevA.36.1396.

(13) Baran, D.; Balan, A.; Celebi, S.; Meana Esteban, B.; Neugebauer, H.; Sariciftci, N. S.; Toppare, L. Processable Multipurpose Conjugated Polymer for Electrochromic and Photovoltaic Applications. https://doi.org/10.1021/cm100372t.

(14) Paul, A.; Borrelli, R.; Bouyanfif, H.; Gottis, S.; Sauvage, F. Tunable Redox Potential, Optical Properties, and Enhanced Stability of Modified Ferrocene-Based Complexes. *ACS Omega* 2019, *4* (12), 14780–14789. https://doi.org/10.1021/ACSOMEGA.9B01341/.

(15) Hansen, W. N.; Kolb, D. M. The Work Function of Emersed Electrodes. *J Electroanal Chem Interfacial Electrochem* 1979, *100* (1–2), 493–500. https://doi.org/10.1016/S0022-0728(79)80180-1.

(16) Takahashi, A.; Kurahashi, T.; Fujii, H. Redox Potentials of Oxoiron(IV) Porphyrin π-Cation Radical Complexes: Participation of Electron Transfer Process in Oxygenation Reactions. *Inorg Chem* 2011, *50* (15), 6922–6928. https://doi.org/10.1021/IC102564E.

(17) Gomer, R.; Tryson, G. An Experimental Determination of Absolute Half-cell Emf's and Single Ion Free Energies of Solvation. *J Chem Phys* 1977, *66* (10), 4413–4424. https://doi.org/10.1063/1.433746.

(18) Zhao, H.; Pan, Y.; Lau, K.-C. Ferrocene/Ferrocenium, Cobaltocene/ Cobaltocenium and Nickelocene/Nickelocenium: From Gas Phase Ionization Energy to One-Electron Reduction Potential in Solvated Medium †. *Phys. Chem. Chem. Phys* 2023, *25*, 16921. https://doi.org/10.1039/d3cp01904g.

(19) Kötz, E. R.; Neff, H.; Müller, K. A UPS, XPS and Work Function Study of Emersed Silver, Platinum and Gold Electrodes. *J Electroanal Chem Interfacial Electrochem* 1986, *215* (1–2), 331–344. https://doi.org/10.1016/0022-0728(86)87026-7.

(20) Namazian, M.; Lin, C. Y.; Coote, M. L. Benchmark Calculations of Absolute Reduction Potential of Ferricinium/Ferrocene Couple in Nonaqueous Solutions. *J Chem Theory Comput* 2010, *6* (9), 2721–2725. https://doi.org/10.1021/CT1003252/.

(21) Cardona, C. M.; Li, W.; Kaifer, A. E.; Stockdale, D.; Bazan, G. C. Electrochemical Considerations for Determining Absolute Frontier Orbital Energy Levels of Conjugated Polymers for Solar Cell Applications. *Advanced Materials* 2011, *23* (20), 2367–2371. https://doi.org/10.1002/adma.201004554.

(22) de Leeuw, D. M.; Simenon, M. M. J.; Brown, A. R.; Einerhand, R. E. F. Stability of N-Type Doped Conducting Polymers and Consequences for Polymeric Microelectronic Devices. *Synth Met* 1997, *87* (1), 53–59. https://doi.org/10.1016/S0379-6779(97)80097-5.

(23) Pommerehne, J.; Vestweber, H.; Guss, W.; Mahrt, R. F.; Bässler, H.; Porsch, M.; Daub, J. Efficient Two Layer Leds on a Polymer Blend Basis. *Advanced Materials* 1995, *7* (6), 551–554. https://doi.org/10.1002/adma.19950070608.

(24) Bredas, J. L. Mind the Gap! *Mater Horiz* 2014, *1* (1), 17–19. https://doi.org/10.1039/c3mh00098b.

(25) Makoś, M. Z.; Gurunathan, P. K.; Raugei, S.; Kowalski, K.; Glezakou, V. A.; Rousseau, R. Modeling Absolute Redox Potentials of Ferrocene in the Condensed Phase. *Journal of Physical Chemistry Letters* 2022, *13* (42), 10005–10010. https://doi.org/10.1021/ACS.JPCLETT.2C02447/

(26) Isse, A. A.; Gennaro, A. Absolute Potential of the Standard Hydrogen Electrode and the Problem of Interconversion of Potentials in Different Solvents. *Journal of Physical Chemistry B* 2010, *114* (23), 7894–7899. https://doi.org/10.1021/JP100402X/.

(27) Bard, A. J.; Faulkner, L. R. *Electrochemical Methods : Fundamentals and Applications*; Wiley, 2001.

(28) Baik, M. H.; Friesner, R. A. Computing Redox Potentials in Solution: Density Functional Theory as a Tool for Rational Design of Redox Agents. *Journal of Physical Chemistry A* 2002, *106* (32), 7407–7412. https://doi.org/10.1021/JP025853N/.

(29) Trasa'tti, S. The Absolute Electrode Potential: An Explanatory Note (Recommendations 1986). *Pure and Applied Chemistry* 1986, *58* (7), 955–966. https://doi.org/10.1351/PAC198658070955/.

(30) Castro, L.; Bühl, M. Calculations of One-Electron Redox Potentials of Oxoiron(IV) Porphyrin Complexes. *J Chem Theory Comput* 2014, *10* (1), 243–251. https://doi.org/10.1021/CT400975W.

(31) Fawcett, W. R. The Ionic Work Function and Its Role in Estimating Absolute Electrode Potentials. *Langmuir* 2008, *24* (17), 9868–9875. https://doi.org/10.1021/LA7038976.

(32) Winget, P.; Weber, E. J.; Cramer, C. J.; Truhlar, D. G. Computational Electrochemistry: Aqueous One-Electron Oxidation Potentials for Substituted Anilines. *Physical Chemistry Chemical Physics* 2000, *2* (6), 1231–1239. https://doi.org/10.1039/A909076B.

(33) Toma, M.; Kuvek, T.; Vrč, V. Ionization Energy and Reduction Potential in Ferrocene Derivatives: Comparison of Hybrid and Pure DFT Functionals. *J. Phys. Chem* 2020, *2020*, 8029–8039. https://doi.org/10.1021/acs.jpca.0c06663.

(34) Gleiter, R.; Bohm, M. C.; Ernst, R. D. The He(I) Photoelectron Spectrum of Bis(Pentadienyl) Iron: A Comparison with the Ferrocene Spectrum. *J Electron Spectros Relat Phenomena* 1984, *33* (3), 269–278. https://doi.org/10.1016/0368-2048(84)80023-7.

(35) Roy, L. E.; Jakubikova, E.; Graham Guthrie, M.; Batista, E. R. Calculation of One-Electron Redox Potentials Revisited. Is It Possible to Calculate Accurate Potentials with Density Functional Methods? *Journal of Physical Chemistry A* 2009, *113* (24), 6745–6750. https://doi.org/10.1021/JP811388W.

(36) Wang F, Vasilyev V. Fe 3d Orbital Evolution in Ferrocene Ionization: Insights from ΔSCF, EOES, and Orbital Momentum Distribution. Molecules. 2025 Aug 29;30(17):3541. doi: 10.3390/molecules30173541.

(37) Barfuss, S.; Grade, M.; Hirschwalde, W.; Rosinger, W.; Boag, N. M.; Driscoll, D. C.; Dowben, P. The Stability and Decomposition of Gaseous Chloroferrocenes. *Peter Dowben Publications* 1987.

(38) Begun, G. M.; Compton, R. N. Electron Impact Ionization Studies of Ferrocene, Cobaltocene, Nickelocene, and Magnesocene. *J Chem Phys* 1973, *58* (6), 2271–2280. https://doi.org/10.1063/1.1679502.

(39) Opitz, J.; Härter, P. Multiphoton Ionization of Vanadocene and Ferrocene at 248 and 193 Nm. Wavelength-Dependent Competition between Dissociation and Ionization. *Int J Mass Spectrom Ion Process* 1992, *121* (3), 183–199. https://doi.org/10.1016/0168-1176(92)80062-6.

(40) Meot-Ner, M. Ion Chemistry of Ferrocene. Thermochemistry of Ionization and Protonation and Solvent Clustering. Slow and Entropy-Driven Proton-Transfer Kinetics. *J. J. Electron Spectrosc: Theory, Tech. Appl* 1989, *111* (2), 2956.

(41) Ryan, M. F.; Eyler, J. R.; Richardson, D. E. Adiabatic Ionization Energies, Bond Disruption Enthalpies, and Solvation Free Energies for Gas-Phase Metallocenes and Metallocenium Ions. *J. Am. Chem. Soc* 1992, *114*, 8611–8619.

(42) Rabalais, J. W.; Werme, L. O.; Bergmark, T.; Karlsson, L.; Hussain, M.; Siegbahn, K. Electron Spectroscopy of Open-Shell Systems: Spectra of Ni(C5H5)2, Fe(C5H5)2, Mn(C5H5)2, and Cr(C5H5)2. *J Chem Phys* 1972, *57* (3), 1185–1192. https://doi.org/10.1063/1.1678375.

(43) Lu, J.; Nagase, S.; Yu, D.; Ye, H.; Han, R.; Gao, Z.; Zhang, S.; Peng, L. Amphoteric and Controllable Doping of Carbon Nanotubes by Encapsulation of Organic and Organometallic Molecules. *Phys Rev Lett* 2004, *93* (11). https://doi.org/10.1103/PHYSREVLETT.93.116804.

(44) Altwegg, L.; Pope, M.; Arnold, S.; Fowlkes, W. Y.; El Hamamst, M. A. Electrostatic Determination of Photo-Ionization Potentials of Solids and Liquids. *Autoionization in Anthracene The Journal of Chemical Physics* 1982, *53*, 2197. https://doi.org/10.1063/1.1136958.

(45) Delahay, P. Photoelectron Emission Spectroscopy of Aqueous Solutions. *Acc. Chem. Res* 1982, *15*, 40–45.

(46) Ishii, H.; Kinjo, H.; Sato, T.; Machida, S. I.; Nakayama, Y. Photoelectron Yield Spectroscopy for Organic Materials and Interfaces. *Electronic Processes in Organic Electronics: Bridging Nanostructure, Electronic States and Device Properties* 2015, 131–155. https://doi.org/10.1007/978-4-431-55206-2_8.

(47) Gurevich, Yu. Ya.; Pleskov, Yu. V.; Rotenberg, Z. A. *Photoelectrochemistry*; 1967.

(48) Gale, R. *Spectroelectrochemistry Theory and Practice*; 1971; Vol. 5.

(49) Watanabe, I.; Flanagan, J. B.; Delahay, P. Vacuum Ultraviolet Photoelectron Emission Spectroscopy of Water and Aqueous Solutions. *J Chem Phys* 1980, *73*, 174506. https://doi.org/10.1063/1.440427.

(50) Brodsky, A. M.; Tsarevsky, A. V. Emission of Electrons from Solutions.

(51) Brodskii, A. M.; Gurevich, Y. Y.; Levich, V. G. General Threshold Theory of Electronic Emission from the Surface of a Metal. *physica status solidi (b)* 1970, *40* (1), 139–151. https://doi.org/10.1002/PSSB.19700400115.

(52) Brodsky, A. M. An Investigation of the Photoelectron Emission from Solutions Containing Solvated Electrons, and the Physical Nature of the Solvated Electron. *Journal of Physical Chemistry* 1980, *84* (14), 1856–1863. https://doi.org/10.1021/J100451A024.

(53) Gurevich, Y. Y.; Krotova, M. D.; Pleskov, Y. V. Electron Photoemission from Semiconductors into Electrolyte Solutions. *J Electroanal Chem Interfacial Electrochem* 1977, *75* (1), 339–351. https://doi.org/10.1016/S0022-0728(77)80092-2.

(54) Baron, B.; Delahay, P.; Lugo, R. Thermionic Emission by Solutions of Solvated Electrons. *J Chem Phys* 1970, *53* (4), 1399–1405. https://doi.org/10.1063/1.1674186.

(55) Mott.N F., E. A. D. Electronic Process in Non-Crystalline Materials. 2012, 605.

(56) Sommerfeld, A. Über Die Ausbreitung Der Wellen in Der Drahtlosen Telegraphie. *Ann Phys* 1909, *333* (4), 665–736. https://doi.org/10.1002/ANDP.19093330402.

(57) Benderskii, V. A.; Grebenshchikov, S. Y. Photoemission from Metals and the Slowing of Low Energy Electrons in Water. *Journal of Electroanalytical Chemistry* 1993, *358* (1–2), 111–125. https://doi.org/10.1016/0022-0728(93)80433-I.

(58) Gremmo, N.; Randles"~, J. E. B. Solvated Electrons in Hexamethylphosphoramide Part 2.- Density Measurements, Electrical Properties of Frozen Solutions, Electron Emission from Surfaces of Solutions. 1974.

(59) Jost, R. Uber Die Falschen Nullstellen Der Eigenwerte Der S-Matrix. *Helv. Phys. Acta* 1947, *20*, 256–266.

(60) De Alfaro, V. (Vittorio); Regge, T. *Potential Scattering / V. de Alfaro and T. Regge*; North-Holland: Amsterdam, 1965.

(61) Pleskov, Y. V; Rotenberg, Z. A. *The Photoelectric Effect at the Metal-Electrolyte Boundary Related Content Photoemission of Electrons from Metals into Electrolyte Solutions*.

(62) Rotenberg, Z. A.; Lakomov, V. I.; Pleskov, Y. V. Electron Photoemission as a New Method for Studying the Electric Double Layer Structure and the Kinetics of Electrochemical Reactions. *J Electroanal Chem Interfacial Electrochem* 1970, *27* (3), 403–419. https://doi.org/10.1016/S0022-0728(70)80236-4.

(63) D, Y.; A, I. Characterization of Catechins in Water by Photoemission Yield Spectroscopy in Air. *Anal Sci* 2016, *32* (5), 577–580. https://doi.org/10.2116/ANALSCI.32.577.

(64) Von Burg, K.; Delahay, P. Electron Transfer Reactions in Photoelectron Emission Spectroscopy of Aqueous Solutions. *Chem Phys Lett* 1981, *83* (1), 199–203. https://doi.org/10.1016/0009-2614(81)80319-3.

(65) Delahay, P.; Von Burg, K.; Dziedzic, A. Photoelectron Emission Spectroscopy of Inorganic Cations in Aqueous Solution. *Chem Phys Lett* 1981, *79* (1), 157–161. https://doi.org/10.1016/0009-2614(81)85309-2.

(66) Delahay, P. Charge Transfer Spectra and Photoelectron Emission by Solutions. *Chem Phys Lett* 1982, *89* (2), 149–153. https://doi.org/10.1016/0009-2614(82)83392-7.

(67) Petersen, P. B.; Saykally, R. J. Probing the Interfacial Structure of Aqueous Electrolytes with Femtosecond Second Harmonic Generation Spectroscopy. *Journal of Physical Chemistry B* 2006, *110* (29), 14060–14073. https://doi.org/10.1021/JP0601825.

(68) Weber, R. Photoelectron Spectroscopy of Liquid Water and Aqueous Solutions in Free Using Synchrotron Radiation. 2003. https://doi.org/10.17169/REFUBIUM-5706.

(69) Ottosson, N.; Faubel, M.; Bradforth, S. E.; Jungwirth, P.; Winter, B. Photoelectron Spectroscopy of Liquid Water and Aqueous Solution: Electron Effective Attenuation Lengths and Emission-Angle Anisotropy. *J Electron Spectros Relat Phenomena* 2010, *177* (2–3), 60–70. https://doi.org/10.1016/J.ELSPEC.2009.08.007.

(70) Nitzan, A. Electron Transmission through Molecules and Molecular Interfaces. *Annu Rev Phys Chem* 2001, *52* (Volume 52, 2001), 681–750. https://doi.org/10.1146/ANNUREV.PHYSCHEM.52.1.681

(71) Stephens, J. A.; Fano, U. Slow Electrons in Condensed Matter: The Large Polaron. *Phys Rev A (Coll Park)* 1988, *38* (7), 3372. https://doi.org/10.1103/PhysRevA.38.3372.

(72) Fano, U.; Stephens, J. A. Slow Electrons in Condensed Matter. *Phys Rev B* 1986, *34* (1), 438. https://doi.org/10.1103/PhysRevB.34.438.

(73) Nguyen-Truong, H. T. Low-Energy Electron Inelastic Mean Free Paths for Liquid Water. *Journal of Physics: Condensed Matter* 2018, *30* (15), 155101. https://doi.org/10.1088/1361-648X/AAB40A.

(74) Lee, A. A.; Perez-Martinez, C. S.; Smith, A. M.; Perkin, S. Scaling Analysis of the Screening Length in Concentrated Electrolytes. *Phys Rev Lett* 2017, *119* (2), 026002. https://doi.org/10.1103/PHYSREVLETT.119.026002.

(75) Adar, R. M.; Safran, S. A.; Diamant, H.; Andelman, D. Screening Length for Finite-Size Ions in Concentrated Electrolytes. *Phys Rev E* 2019, *100* (4), 042615. https://doi.org/10.1103/PHYSREVE.100.042615.

(76) Smith, A. M.; Lee, A. A.; Perkin, S. The Electrostatic Screening Length in Concentrated Electrolytes Increases with Concentration. *Journal of Physical Chemistry Letters* 2016, *7* (12), 2157–2163. https://doi.org/10.1021/ACS.JPCLETT.6B00867

(77) Woods, E.; Konys, C. A.; Rossi, S. R. Photoemission of Iodide from Aqueous Aerosol Particle Surfaces. 2019. https://doi.org/10.1021/acs.jpca.8b12323.

(78) Salmeron, M.; Schlögl, R. Ambient Pressure Photoelectron Spectroscopy: A New Tool for Surface Science and Nanotechnology. *Surf Sci Rep* 2008, *63* (4), 169–199. https://doi.org/10.1016/J.SURFREP.2008.01.001.

(79) Groß, A.; Sakong, S. Ab Initio Simulations of Water/Metal Interfaces. *Chem Rev* 2022, *122* (12), 10746–10776. https://doi.org/10.1021/ACS.CHEMREV.1C00679/

(80) Becker, M.; Loche, P.; Rezaei, M.; Wolde-Kidan, A.; Uematsu, Y.; Netz, R. R.; Bonthuis, D. J. Multiscale Modeling of Aqueous Electric Double Layers. *Chem Rev* 2024, *124* (1), 1–26. https://doi.org/10.1021/ACS.CHEMREV.3C00307/

(81) Emelyanenko, K. A.; Emelyanenko, A. M.; Boinovich, L. Image-Charge Forces in Thin Interlayers Due to Surface Charges in Electrolyte. *Phys Rev E Stat Nonlin Soft Matter Phys* 2015, *91* (3), 032402. https://doi.org/10.1103/PHYSREVE.91.032402/.

(82) Souna, A. J.; Motevaselian, M. H.; Polster, J. W.; Tran, J. D.; Siwy, Z. S.; Aluru, N. R.; Fourkas, J. T. Beyond the Electrical Double Layer Model: Ion-Dependent Effects in Nanoscale Solvent Organization. *Physical Chemistry Chemical Physics* 2024, *26* (8), 6726–6735. https://doi.org/10.1039/D3CP05712G.

(83) Polster, J. W.; Souna, A. J.; Motevaselian, M. H.; Lucas, R. A.; Tran, J. D.; Siwy, Z. S.; Aluru, N. R.; Fourkas, J. T. The Electrical-Double Layer Revisited. *Natural Sciences* 2022, *2* (2), e20210099. https://doi.org/10.1002/NTLS.20210099.

(84) Delahay, P.; Dziedzic, A. Gas—Liquid Correlation of Ionization Energies. *Chem Phys Lett* 1984, *108* (2), 169–173. https://doi.org/10.1016/0009-2614(84)85714-0.

(85) Delahay, P.; Dziedzic, A.; Appl Phys, J.; Delahay, P.; Dziedzic, A. Dispersion Spectroscopy of Optical Electron Transfer in Solution. *J Chem Phys* 1984, *80* (11), 5381–5387. https://doi.org/10.1063/1.446669.

(86) Krishtalik, L. I.; Alpatova, N. M.; Ovsyannikova, E. V. Determination of the Surface Potentials of Solvents. *Journal of Electroanalytical Chemistry* 1992, *329* (1–2), 1–8. https://doi.org/10.1016/0022-0728(92)80204-H.

(87) Randles, J. E. Structure at the Free Surface of Water and Aqueous Electrolyte Solutions. *Phys Chem Liquids* 1977, *7* (1–2), 107–179. https://doi.org/10.1080/00319107708084730.
